# Review of Current International Decision-Making Processes for Newborn Screening: Lessons for Australia

**DOI:** 10.3389/fpubh.2015.00214

**Published:** 2015-09-10

**Authors:** Selina Carolyne Metternick-Jones, Karla Jane Lister, Hugh J. S. Dawkins, Craig Anthony White, Tarun Stephen Weeramanthri

**Affiliations:** ^1^Office of Population Health Genomics, Public Health Division, Department of Health, Government of Western Australia, Perth, WA, Australia; ^2^School of Medicine, University of Tasmania, Hobart, TAS, Australia; ^3^Public Health Division, Department of Health, Government of Western Australia, Perth, WA, Australia

**Keywords:** newborn bloodspot screening, policy, public health, screening, decision-making

## Abstract

Newborn bloodspot screening has been operating successfully in Australia for almost 50 years. Recently, the development of new technologies and treatments has led to calls for the addition of new conditions to the screening programs. Internationally, it is recognized by governments that national policies for newborn screening should support transparent and evidence-based decision making, and promote consistency between states within a country. Australia is lagging behind the international community, and currently has no national policies or decision-making processes, agreed by government, to support its newborn screening programs. In contrast, New Zealand (NZ), the United Kingdom (UK), and the United States of America (US) have robust and transparent processes to assess conditions for screening, which have been developed by, and have pathways to, government. This review provides detail on the current policy environment for newborn screening in Australia, highlighting that there are a number of risks to the programs resulting from the lack of a decision-making process. It also describes the processes used to assess conditions for newborn screening in the US, UK, and NZ. These examples highlight the benefits of developing a national decision-making process, including ensuring that screening is evidence based and effective. These examples also provide models that might be considered for Australia, as well as other countries currently seeking to introduce or expand newborn bloodspot screening.

## Introduction

Newborn bloodspot screening is an important public health program that has been operating globally since the 1960s. It aims to screen all newborns to detect a range of potentially life-threatening conditions before symptoms present. Typically, all of the conditions for which screening is offered benefit from early intervention, with treatment often preventing physical and mental disability, and death (1). Due to differences in government policy, structure, and priorities, the number of conditions screened varies dramatically around the world, with some countries providing no newborn bloodspot screening, and others screening for more than 40 conditions (2).

There are many conditions, in addition to those currently screened, which could be included in newborn screening programs. This is due to ongoing identification of new tests and treatments, which make screening for more conditions technically possible. For example, prior to 2005, severe combined immunodeficiency (SCID) could not be included in newborn screening as there was no method of testing for the condition using a dried bloodspot. With the development of a successful method for bloodspot screening for SCID, a number of pilot studies were conducted. Some countries, including the US, now recommend the condition for inclusion in newborn screening programs (3). The development of new tests, such as the one for SCID, has led to calls to expand current programs, and it is likely that such calls will be ongoing. This is particularly relevant as genomic technologies, which can screen for a vast number of conditions, are becoming more readily available in the clinical setting (4–6).

When considering new conditions for screening, there is a need to ensure that it continues to benefit the individual, community, and health system as well as minimize harm (7). Therefore, the decision to add or remove conditions to newborn screening programs should only be made after a careful assessment of the evidence about benefits and risks. In order to facilitate such an assessment, a methodological decision-making process is required. A number of governments around the world have developed such frameworks to ensure the appropriateness and effectiveness of screening.

The criteria used to assess conditions for screening around the world are based on the Wilson and Jungner principles of early disease detection, included in Box 1 (8). These principles were adopted by the World Health Organization (WHO) and, while considered the gold standard, have been criticized for being ill-defined and too theoretical (9). In most instances, governments have adapted the WHO criteria to suit the local environment and include more detail specific to newborn screening (10). The adaptations have resulted in varying decision-making criteria and subsequent discrepancies in the number of conditions screened between countries. For example, the United States (US) recommends screening for 29 conditions while the United Kingdom (UK) screens for nine, with four of those conditions only becoming part of the UK’s screening program in early 2015. In addition to differences in the screening criteria, there are differences in the extent that these processes have been tested over time. For example, the decision-making process in the UK is well established and has been in place since 2009, whereas the most recent US process was only formalized in April 2013. While there are many international examples of decision-making processes for newborn screening, this review focuses on those from New Zealand (NZ), US, and UK as they are the most relevant to Australia.

Box 1 **WHO principles of early disease detection (8).**

**Condition**

- The condition should be an important health problem.
- There should be a recognisable latent or early symptomatic stage.
- The natural history of the condition, including development from latent to declared disease should be adequately understood.

**Test**

- There should be a suitable test or examination.
- The test should be acceptable to the population.

**Treatment**

- There should be an accepted treatment for patients with recognised disease.

**Screening Program**

- There should be an agreed policy on whom to treat as patients.
- Facilities for diagnosis and treatment should be available.
- The cost of case-findings (including diagnosis and treatment of patients diagnosed) should be economically balanced in relation to possible expenditure on medical care as a whole.
- Case-findings should be a continuing process and not a ‘once and for all’ project.

## The Australian Setting

Newborn screening has been operating successfully in Australia for almost 50 years across the six federated states and two territories. More than 99% of all babies are screened for around 25 genetic and metabolic conditions (11, 12). In Australia, newborn screening programs are funded by state and territory governments and operate independently of each other. This funding model means each state and territory makes its own decisions regarding screening, including which conditions to screen. In the past half century, the programs have undergone significant change, growing from an initiative, which screened for a single condition, phenylketonuria, to the programs we see today. Despite these changes, there has been no specific policy or process, agreed by governments, to guide decisions regarding which conditions should be screened.

In Australia, changes to newborn screening programs have been made in an *ad hoc* manner, usually reactively and often in response to new technologies. Historically, one state or territory has implemented a new technology, or begun screening for an additional condition, and others have followed suit. The key example of this was the introduction in the late 1990s of tandem mass spectrometry in screening laboratories, which was followed by an expansion in the conditions screened. This technology enables simultaneous testing for a vast number of conditions. New South Wales piloted the technology in 1998, followed by South Australia in 1999. Screening with tandem mass spectrometry became universal in Australia in 2005, with other states adopting the technology as they could secure funding for the equipment.

The introduction of tandem mass spectrometry resulted in the addition of around 20 new conditions to screening protocols. The decision to implement new technology was supported by the Royal Australasian College of Physicians and the Human Genetics Society of Australasia. However, governments did not follow a transparent decision-making process to assess the evidence regarding benefits and harms of screening for individual conditions. Instead, decisions were made in response to the developing inequalities in the conditions screened between states, with technology as the initial driver.

The expanded screening programs, which emerged following the implementation of tandem mass spectrometry were, and remain, relatively stable and consistent. The key exception is the Victorian laboratory which does not screen for galactosemia. It can be argued, with considerable merit, that the high level of consistency is due to the dedication and professionalism of the newborn screening program leaders and the strong professional networks that exist. It can also be argued that an approach, which relies so strongly on internal drivers, is not sustainable to support ongoing decision making within and about programs. This is evidenced through a decade-long proposal to have congenital adrenal hyperplasia (CAH), a rare and potentially fatal condition, assessed for inclusion in newborn screening programs (4, 13). Despite significant efforts from those within the programs to have CAH assessed, the issue has been referred back and forth among state, territory, and Commonwealth governments without any decision being made (14, 15). This is largely due to the lack of an agreed decision-making process fit for this purpose, indicating that the current approach falls short of what is now required.

Since newborn screening was last expanded in the early 2000s, Australia’s commitment to population-based screening has evolved considerably. There is now recognition of the national structures, policies, and processes required to support effective and efficient population-based screening. Central to this has been the establishment of the Australian Screening Advisory Committee in 2001, now known as the Standing Committee on Screening (SCoS). This committee is tasked with providing guidance to the national cancer screening programs and considers emerging screening issues. SCoS comprises senior health officials, with expertise in screening, from the state, territory, and Commonwealth health departments.

Historically, SCoS and its former iterations have focused primarily on the national screening programs for breast, cervical, and bowel cancer. These programs are funded either solely by the Commonwealth Government or through partnership with state and territory governments. In addition to SCoS’s focus on cancer screening programs, it also considers emerging screening issues of national significance. It has developed national policies for newborn hearing screening, reviewed the evidence for lung cancer screening and released a position statement on prostate cancer screening (16). Despite this, SCoS has never been involved in providing policy guidance for newborn screening. However, this has recently changed. In the past year, SCoS has recognized the importance of national guidance for newborn screening and is now in the early stages of developing a national policy framework for newborn screening, which will include a decision-making framework (17).

In Australia, SCoS has played an important role in providing guidance on what constitutes a good screening program through the development of the *Population-Based Screening Framework* in 2008 (Box 2) (7). The framework is based on the WHO Principles and has been endorsed by the Australian Health Ministers’ Advisory Council, with the aim of guiding decision makers when implementing screening programs (7). The development of this framework highlights that Australian governments recognize the importance of clear decision-making processes and policies for population-based screening programs. While it does provide some high level guidance, it lacks specific and relevant detail to enable an assessment of rare genetic conditions for newborn screening. For this reason, the framework is not sufficient to act as the sole decision-making guide for newborn screening.

Box 2 **Australian population-based screening framework: screening program requirements (7).**

**The Screening Program must:**

- respond to a recognised need.
- have a clear definition of the objectives of the programand the expected health benefits.
- have scientific evidence of screening program effectiveness.
- identify the target population which stands to benefit from screening.
- clearly define the screening pathway and interval.
- ensure availability of the organisation, infrastructure, facilities and workforce needed to deliver the screening program.
- have measures available that have been demonstrated to be cost effective to encourage high coverage.
- have adequate facilities available for having tests and interpreting them.
- have an organised quality control program across the screening pathway to minimise potential risks of screening.
- have a referral system for management of any abnormalities found and for providing information about normal screening tests.
- have adequate facilities for follow-up assessment, diagnosis, management and treatment.
- have evidence based guidelines and policies for assessment, diagnosis and support for people with a positive test result.
- have adequate resources available to set up and maintaina database of health information collected for the program.
- integrate education, testing, clinical services and program management.
- have a database capable of providing a population register for people screened that can issue invitations for initial screening, recall individuals for repeat screening, follow those with identified abnormalities, correlate with morbidity and mortality results and monitor and evaluate the program and its impact.
- plan evaluation from the outset and ensure that program data are maintained so that evaluation and monitoring of the program can be performed regularly.
- be cost-effective.
- ensure informed choice, confidentiality and respect for autonomy.
- promote equity and access to screening for the entire target population.
- ensure the overall benefits of screening outweigh the harm.

In 2011, the Human Genetics Society of Australasia, in conjunction with the Division of Paediatrics of the Royal Australasian College of Physicians, released its policy recommendations for newborn screening (1). These recommendations stressed that there is an “urgent need for the development of a national evidence-based process to evaluate proposals for changes to the conditions covered by newborn screening programs” (1). The policy includes a tool to assess the appropriateness of conditions for newborn screening in the Australian context, and a list of conditions recommended for screening. This is the sole example of national decision-making guidance for newborn screening in Australia. However, it has not been adopted by governments and, hence, does not translate into policy.

It is apparent that newborn screening in Australia is operating in an environment, which lacks a considered decision-making process for government, particularly in regards to assessing conditions for screening. This poses a risk to the programs as it could result in conditions not being thoroughly assessed in a consistent manner across Australia, prior to implementation – or exclusion. This may lead to unnecessary harms for parents and babies, including increased anxiety, as a result of false positive results or indeterminate results of uncertain significance (18). The past *ad hoc* and technology-driven approach is no longer appropriate, and is unable to produce the transparent and carefully balanced decisions that programs require. Changes to the programs should be informed by the result of a robust process that allow evidence about the benefits and harms of screening to be carefully considered. This will ensure that screening is informed by the best possible evidence, provides the greatest utility and is driven by what is best for the newborn (19). While professional networks have recognized this need, governments have been slower to respond, and thus decision making for the programs has stalled.

## International Experiences

Decisions to expand newborn screening programs have historically been determined by “advances in technology, medical opinion, and pressure from interest groups, rather than through an evidence-based process” (20). In contrast, the international examples explored in this review show that having a cohesive process to manage the introduction of new conditions ensures an evidence-based approach, and promotes consistency and transparency across jurisdictions (19, 20).

### New zealand

In NZ, the National Screening Unit (NSU) is responsible for the “safety, effectiveness, and quality of organized screening programs,” including the Newborn Metabolic Screening Programme. The NSU is responsible for funding, contracting, auditing, and monitoring of services as well as policy development, guidelines, and consumer resources. It contracts one screening laboratory that is responsible for the testing and reporting of samples, which makes its structure notably different from Australia. The NSU also works closely with maternity services, the screening laboratory, and the metabolic clinical team to ensure screening is timely and of high quality.

Prior to the development of a decision-making framework to assess new conditions for newborn screening in 2011, NZ expanded its program to include a number of rare metabolic conditions. This expansion came with the implementation of tandem mass spectrometry in 2006, after the equipment was gifted to the program by a charitable organization (21). The resulting expansion was spurred by evidence indicating that metabolic disorders were being underdiagnosed in NZ (22).

In 2011, the NSU published the *Newborn Metabolic Screening Programme Policy Framework*, which outlined national policies, including the process for assessing conditions suitable for population-based newborn screening (23). The decision-making process involves the nomination of a condition, either by a health professional, an interested organization, a patient support group or a member of the public. If the NSU deems there is sufficient evidence to support further consideration, the nominated condition is assessed by the Programme Governance Team.

The Governance Team may request further information, such as an evidence review, cost benefit analysis, analysis against the screening criteria, and/or stakeholder consultation. The Programme Governance Team then provides advice to the NSU for or against inclusion. The next phase may involve pilot studies as well as documentation of treatment and laboratory protocols before the NSU begins implementing routine screening for the recommended condition.

The decision-making framework is currently being used by the NSU to evaluate SCID for inclusion in its program. The proposal to assess SCID was developed by immunology clinicians before being submitted to the NSU (24). The fact that the program is reviewing new conditions for screening highlights the capacity of evidence-based decision making to enable rational changes to newborn screening. The Policy Framework also allows for conditions to be considered for removal from the program. The process enables the program to respond to new and emerging evidence, and ensure NZ’s families have access to the most appropriate newborn screening.

### United states

In the US, like Australia, each state is responsible for funding and implementing its own newborn screening program. This led to inconsistencies in the conditions screened between states, with some screening for as few as three conditions and others mandating as many as 43 (19). In 2002, in response to these inconsistencies, the Health Resources and Services Administration, and the American Academy of Pediatrics tasked the American College of Medical Genetics (ACMG) with developing a core panel of conditions that should be screened across the US. A total of 84 potential conditions were evaluated before a core panel of 25 conditions was recommended (19).

To evaluate the 84 conditions for newborn screening, the ACMG formed a working group, which included experts in various subspecialties of medicine and primary care, health policy, law, ethics, and public health as well as consumers. The working group used a two-tiered approach for assessing conditions (19). The first tier involved conducting a survey to collect information on the condition and allow for input from individuals and organizations with an interest in screening. The second tier of analysis involved establishing a strong evidence base for assessing the condition, including gathering scientific knowledge related to the test and treatment. From this information, the working group determined whether each condition should be recommended as either: (a) part of the core panel, (b) a secondary target, or (c) excluded from screening.

The recommendations of the ACMG for a core panel of conditions were agreed by the Health Resources and Services Administration, and subsequently newborn screening was expanded. While this process did lead to a national recommendation to screen for a consistent range of conditions, it has been criticized by the US Preventative Services Task Force for not being in line with accepted standards for evidence-based decision making. The Task Force indicated that “the ACMG’s approach … relied mostly on colloquial evidence” (25) and that there had not been an appropriate level of engagement with the community.

In order to provide a more transparent, consistent, and ongoing mechanism to assess conditions for newborn screening, the Discretionary Advisory Committee on Heritable Disorders in Newborns and Children (the committee) was established in 2013 (26). This committee and its operations are funded by the Federal Department of Health and Human Services. The process used by the committee to assess conditions involves an evidence review conducted by an external group. This review summarizes evidence from both published and unpublished sources on the benefits and harms of screening for the nominated condition (27). Using this evidence, the committee assesses both the magnitude and the certainty of net benefits from screening (28). This includes weighing health benefits to the newborn against the harms, and the effectiveness of screening against usual clinical care (27).

In addition to analyzing the benefits of screening, the committee makes an assessment of states’ readiness to implement screening for the nominated condition. This includes states’ capacity to introduce screening and support both short- and long-term follow up of babies diagnosed with the condition (27). The committee combines the projected net benefits of screening with states’ readiness to screen when making a recommendation to include or exclude a condition from the recommended panel. Following which, recommendations are made to the US Department of Health and Human Services. It is then up to each state to decide when and how to implement screening for the new condition within its program. SCID has been added to the panel using this revised decision-making process, and a number of conditions have been excluded including Pompe disease and Krabbe disease (27).

The assessment of conditions for newborn screening in the US is conducted and funded at the national level. States are then responsible for implementation. However, some national funding is available to support program expansion through grants from the federal government. These funding arrangements are detailed in the *Newborn Screening Saves Lives Act*, which was passed by the US Congress in 2008 and reauthorized in 2014. The purpose of this legislation is to support state newborn screening programs to expand and improve as well as provide national guidance on what conditions should be screened (29).

While the US has a robust decision-making process that provides national recommendations to screen, newborn screening still faces a number of issues at the state level. This comes in the form of local pressure from lobbyists, patient groups, or well-known personalities, calling for screening for additional conditions. An example of this was the introduction of screening for Krabbe disease in New York state. When the ACMG reviewed conditions and developed a national screening panel, they specifically excluded Krabbe disease as treatments are still experimental. Even so, this condition was added to the newborn screening program in New York state after lobbying from a prominent former footballer (30). This highlights that even when decision-making processes are in place, there are still contextual factors, which need to be considered to ensure that the process is robust and holds up to local pressure.

### United kingdom

In the UK, the National Health Service, which is responsible for providing health services to England, Scotland, Wales, and Northern Ireland, established the National Screening Committee (NSC) in 1996. This committee provides advice on the development, implementation, and review of screening programs, including newborn screening (31).

The NSC developed a strict decision-making framework for screening, which is broken down into four key steps. The first step involves identification of key stakeholders, including “patient and professional groups working at a national level” (32). Second, an information scientist conducts a systematic review of the literature, after which the need for external review and consultation is determined. If there is sufficient evidence, an external review is undertaken by an expert in the field as determined by the NSC. The review is then circulated among key stakeholders and the public for their input. Finally, the review is considered by the NSC for endorsement. After endorsement, it is up to each jurisdiction as to how screening for the condition will be implemented.

The NSC decision-making process has been in place since 2009 and was updated in November 2011 (32). To aid the consistency of the process, a comprehensive template for nominating conditions, and for conducting external reviews, is used. It includes a set of 22 criteria which are assessed as met, partially met, not met, or not applicable. Since its implementation, the template has been used to assess a number of conditions for newborn screening including CAH, amino and organic acid metabolism disorders, Duchenne muscular dystrophy, and SCID. None of these conditions have been recommended for screening. However in May 2014, the NSC recommended the addition of four new conditions to newborn screening (33). This is the first addition of conditions in the UK since 2007, and almost doubles the number of conditions screened. This demonstrates that the UK has a more conservative approach to assessing conditions than many other countries (34).

### Other international examples

In addition to the aforementioned processes, a number of other countries have recognized the need for a more transparent approach to assess conditions for newborn screening. Processes exist in the Netherlands (35), Canada (36) and a coordinated multi-national approach has been suggested in Europe. In 2009, the European Union established the European Union Network of Experts on Newborn Screening. This network has completed a detailed review of newborn screening in member countries, and developed a comprehensive set of recommendations, including a proposed model for decision making to assess new conditions (37). While this is not a framework *per se*, it highlights the growing recognition of the importance of a robust decision-making process for newborn screening.

## Discussion

Considered decision making by governments for newborn screening is important to ensure that programs continue to provide high quality screening to newborns. Having an agreed, national, and structured decision-making process means that conditions can be assessed in a transparent and consistent manner. It also means that decisions are based on current evidence. This helps to avoid technology-driven decisions, which do not systematically assess the potential benefits and harms of screening for particular conditions.

A recent examination of newborn bloodspot screening across the globe highlights that in both developed and developing nations, newborn screening programs are either being considered, or on the verge of some form of expansion (38). As such, the current review offers guidance to governments and others to ensure that future developments in these countries are evidence-based and meet the best needs of their populations. Ways in which this guidance can be operationalized is explored through the Australian setting of a federated health system. Therefore, while the following has relevance for all newborn screening settings, it is particularly relevant to those countries where decisions about newborn screening are shared between different levels of government.

The decision-making processes followed in the UK, US, and NZ highlight that, while no one “solution” exists for newborn screening, there are common elements between the different countries. Namely, the decision-making processes within these countries are all:

1. based on the WHO principles, and adapted to suit the local environment;
2. triggered by a condition being nominated for assessment either by government, health professionals or the public;
3. assessed through multidisciplinary stakeholder engagement, to consider the evidence and make recommendations, conducted mostly through committee structures or consultation approaches; and
4. developed by government, with decision making and governance pathways to government.

While the decision-making criteria adhere to the principles recommended by Wilson and Jungner, they differ between countries in the level of detail provided and the way in which evidence is considered against them. The US model includes a quantitative assessment of the evidence and has resulted in a more liberal assessment of conditions (Table 1). Other processes, such as that in the UK, can be considered more subjective (Box 3). These differences have led to variations in the conditions recommended for screening between these countries, with the UK being more conservative than the US. It can be argued that other countries that are introducing or expanding newborn screening should use this knowledge and evidence to find the right balance between these two approaches. Striking this balance would support robust and transparent assessment of conditions, and facilitate evidence-based program development. It would also minimize the extent to which lobby groups could influence decision making.

**Table 1:** **United States decision-making criteria used to assess aspects of the condition for newborn screening (19)**.

Criteria	Categories	Score
Incidence of condition	>1:5,000	100
	>1:25,000	75
	>1:50,000	50
	>1:75,000	25
	<1:100,000	0
Signs and symptoms clinically identifiable in the first 48 h	Never	100
	<25% cases	75
	<50% cases	50
	<75% cases	25
	Always	0
Burden of disease (natural history if untreated)	Profound	50
	Severe	100
	Moderate	75
	Mild	25
	Minimal	0

*The US decision-making framework includes 14 criteria against which a condition is assessed. The following table is an example of three of the criteria. Conditions are scored against the criteria by stakeholders, with data being validated by experts. The mean score given for each criterion by stakeholders is then summed. The maximum possible score for a condition across all 14 criteria is 2100. Any score above 1200 is considered appropriate for inclusion on the recommended core panel for screening*.

Box 3 **United Kingdom decision-making criteria used to assess aspects of the condition for newborn screening (32).**

**The condition:**

1. The condition should be an important health problem.
2. Theepidemiology and natural history should be well understood and there should be a detectable risk factor, disease marker, latent period or early symptomatic stage.
3. All the cost-effective primary prevention interventions should have been implemented asfar as practicable.
4. If carriers of a mutation are identified as a result of screening the natural history of people with this disease, this status should be understood, including the psychological implications.

In many countries, including the US and the UK, newborn screening operates and is managed at a local level. In order to promote a national approach to newborn screening, these countries employ a national or federal agency to make decisions regarding the conditions to be screened. This allows a national recommendation to be made by a respected government body and encourages consistency across states. However, neither the UK nor the US mandates that recommended conditions are adopted by local screening programs. This allows local flexibility to implement screening in line with their budgets and timeframes. A similar structure could be used in the Australian setting as this would allow the state-based programs to maintain autonomy while still having conditions assessed through a transparent and robust process at the national level.

In Australia, as with other countries, funding is one of the biggest barriers to developing and implementing a national decision-making framework for newborn screening. This is because a decision-making framework has funding implications associated with assessing conditions and implementing recommendations. As such, both assessment and implementation require financial support from government. Securing increased funding, from state, territory, or national governments, to support decision making or growth of the programs is difficult in the current fiscal environment. While this presents a potential barrier to progress, there are options for moving forward. There are a number of potential funding models, which could be used to support decision making for newborn screening in Australia, that are also applicable to other programs.

One option with many benefits is for decision making, and the subsequent expansion of a program, to be funded at the national level. In Australia, this responsibility would, therefore, fall upon the Commonwealth government. Centralized decision making and funding would enable a more streamlined transition between a recommendation for screening and its subsequent implementation. However, in Australia funding for newborn screening programs has historically come from state and territory governments, with each state and territory managing its own program. For this reason, it is highly unlikely that the Commonwealth would support such a model.

In contrast to a nationally funded model, a second option would be to have funding for newborn screening decision making and implementation divided between state and national governments. In Australia, this could be achieved through a national committee funded via the Australian Health Ministers’ Advisory Council funding model. Under this model, the Commonwealth, and state and territory governments collectively, each contribute 50% of funds to a shared budget for health activities. A co-funded committee could assess and recommend conditions using a national decision-making process. It would then be up to states and territories to manage, and fund, the implementation of the testing in a manner that suited the local context and budget constraints. This model provides a national recommendation, while still allowing states to have a level of flexibility to implement screening appropriate to their context.

A national decision-making approach, supported by state implementation of decisions, would support consistent decision making across local-level programs. In Australia, this approach also aligns with decision making for other national issues within the health system, such as cancer screening. These initiatives are supported by federal strategic policy but are implemented at the state and territory level. However, even with a national decision-making process, there is still the potential for programs to be influenced by local circumstances. This can occur through lobbying or in response to genuine local differences in health condition prevalence. This was seen with the inclusion of Krabbe disease in the New York state newborn screening program.

A third option to support decision making for newborn screening in countries with regional programs, would be for states to fund both the decision-making process and implementation of any subsequent recommendations. In Australia, this process aligns more closely with the current operations of the program and provides states and territories with greater autonomy. It could be argued that this process is not all that different from the ways in which Australian programs have been expanded in the past: decisions are made individually by the states, and implemented progressively as their budgets permit. As such, careful steps would need to be taken to ensure that this process supports, rather than hinders, consistency of the conditions screened and enables successful ongoing evolution in response to new technology and evidence.

## Conclusion

The efforts undertaken internationally to ensure that newborn screening is appropriate and effective highlight a significant policy gap for Australia. Exploration of these efforts outlines a balanced way forward for decision making in Australia. It also provides guidance for other countries currently considering introducing or expanding newborn screening programs. A uniform framework agreed by governments would not only support consistency into the future, but would provide a transparent mechanism to consider the harms and benefits of screening for new conditions. This approach would help safeguard the programs, which are coming under ever growing pressure to screen for more conditions. This pressure is arising from the rapid growth of new genetic and other diagnostic technologies and novel treatments.

At present in Australia, various professional groups are working together to fill the decision-making gap that exists for newborn screening; however, there is a visible need for a robust process, agreed by governments, to support the consideration of new conditions. Therefore, the road map for success in Australia hinges upon governments coming together to consider the best way forward for local programs. To their credit, Australian Governments have commenced their journey down this path, and are currently drafting a policy framework for newborn bloodspot screening. This policy framework will include a decision-making framework for assessing conditions. While this is commendable, it is only the first step in the process. It is essential that governments agree this framework and support its implementation in order to address the policy gap, and sustain newborn screening programs into the future.

Australia has long been an international leader in the field of newborn screening. However, indecision by governments at the state and national levels, in regards to which conditions to screen, has left Australia lacking reliable safeguards to ensure the continued success of the programs. In the current situation, programs are unable to respond to calls to assess conditions for screening or the appropriateness of new technologies. Agreement must be reached by governments on a decision-making pathway for newborn screening in Australia. Without a national decision-making process for assessing new conditions, Australian newborn screening programs will no longer be able to progress, leaving Australia at real risk of falling behind its international peers – with consequential implications for the health and well-being of its citizens.

## Conflict of Interest Statement

The authors declare that the research was conducted in the absence of any commercial or financial relationships that could be construed as a potential conflict of interest.
